# Next-generation sequencing in liquid biopsy: cancer screening and early detection

**DOI:** 10.1186/s40246-019-0220-8

**Published:** 2019-08-01

**Authors:** Ming Chen, Hongyu Zhao

**Affiliations:** Department of Biostatistics, School of Public Health, Yale University, New Haven, CT USA

**Keywords:** Liquid biopsy, Next-generation sequencing, Cancer screening, Early detection

## Abstract

In recent years, the rapid development of next-generation sequencing (NGS) technologies has led to a significant reduction in sequencing cost with improved accuracy. In the area of liquid biopsy, NGS has been applied to sequence circulating tumor DNA (ctDNA). Since ctDNA is the DNA fragments released by tumor cells, it can provide a molecular profile of cancer. Liquid biopsy can be applied to all stages of cancer diagnosis and treatment, allowing non-invasive and real-time monitoring of disease development. The most promising aspects of liquid biopsy in cancer applications are cancer screening and early diagnosis because they can lead to better survival results and less disease burden. Although many ctDNA sequencing methods have enough sensitivity to detect extremely low levels of mutation frequency at the early stage of cancer, how to effectively implement them in population screening settings remains challenging. This paper focuses on the application of liquid biopsy in the early screening and diagnosis of cancer, introduces NGS-related methods, reviews recent progress, summarizes challenges, and discusses future research directions.

## Introduction

Cancer has a significant impact on public health worldwide. One strategy to lower its burden is through cancer screening and early diagnosis. It is well known that patients have a higher cure rate and 5-year survival if diagnosed at early stages [1]. Medical expense increases dramatically with the stage [2, 3]. Tissue biopsy is the most widely-used tool for cancer detection, staging, and prognosis, but sometimes tumor tissue can be difficult to obtain, especially in metastatic diseases like late-stage lung cancer. Moreover, it is unrealistic to use tissue biopsy for cancer screening and early diagnosis when the tumors have not formed yet. Currently, there are some screening methods proved to be useful for cancer prevention. For example, the mammogram is the best way to detect breast cancer; Pap test is used for early detection of cervical cancer; regular colorectal cancer screening and low-dose computed tomography are recommended to reducing mortality from colorectal cancer and lung cancer, respectively [4]. However, all of these screening methods have limited sensitivity and specificity and are only applicable to a unique cancer type. In order to perform large-scale cancer screening among healthy individuals in the future, a more general and cost-effective approach is needed. In recent years, many scientists and companies have cast their eyes on liquid biopsy [5–8]. Blood contains many types of biological materials like circulating cells, platelets, extracellular vesicles, mRNA, miRNA, protein, and cell-free DNA (cfDNA) [9]. From the blood of cancer patients, a portion of the cfDNA is released by tumor cells through apoptosis, necrosis, or active release [10], and this DNA is called circulating tumor DNA (ctDNA). The tumor-specific mutations in ctDNA sequence can act as a new type of cancer biomarker and help to identify cancer patients from a group of healthy individuals. Compared to traditional cancer diagnosis using tissue biopsy, liquid biopsy is more feasible and less invasive and is more comprehensive than tissue biopsy to evaluate tumor heterogeneity [11] because all tumor sites will release ctDNA into the blood. Facilitated by the rapid development of next-generation sequencing (NGS) technologies, nowadays, ctDNA sequencing can achieve much higher sensitivity than tissue biopsy and can be designed for different purposes [12].

## Applications

### Screening and early diagnosis

Liquid biopsy is a powerful technique that can be applied to different stages of cancer screening and treatment. Among the asymptomatic population, it can be used to identify cancer patients to improve early diagnosis and better intervention. Nevertheless, using ctDNA sequencing for cancer screening and early diagnosis faces great obstacles. Firstly, the concentration of ctDNA is only about 1 to 10 ng/mL in asymptomatic individuals [12]. Therefore, in order to achieve 95% sensitivity, it was shown that around 150 to 300 ml blood sample per test is needed for breast cancer screening [13]. Secondly, apart from the tumor cells, normal healthy cells and hematopoietic cells also contribute to the cfDNA in the blood, resulting in increased false positives when applying ctDNA assays for cancer diagnosis [14]. Great efforts are being made to meet the sensitivity and specificity requirements for cancer screening and early diagnosis [15–17]. Currently, several ctDNA assays can achieve higher sensitivity and specificity than cancer-derived antigens like prostate-specific antigen, carcinoembryonic antigen, carbohydrate antigen (CA) 19-9, CA 15-3, and CA-125 [18]. There are several lines of evidence supporting the further application of ctDNA on screening. Some cohort studies have shown that ctDNA can be used for early lung cancer diagnosis (stage I or II) and can achieve relatively high sensitivity and specificity [16]. Mutations like *KRAS* and *TP53* may be detected in stored sputum samples from individuals up to 1 year before cancer diagnosis [19]. In another prospective study, *KRAS* and *TP53* mutations were detected in the cfDNA of healthy subjects up to 2 years before cancer diagnosis [20]. Apart from DNA mutation, quantification of cfDNA levels [21] and DNA methylation [22] can be combined to provide robust and consistent results. *SEPT9* gene methylation detection is the first US Food and Drug Administration (FDA)-approved blood-based screening test for colorectal cancer (CRC) [23, 24]. It exhibited higher sensitivity and specificity than protein markers [25].

### Treatment selection and prognosis

After cancer diagnosis, ctDNA sequencing enables tumor-specific molecular profile of the patients to guide targeted therapy for precision medicine. The half-life of cfDNA in the circulation is between 16 min and 2.5 h [26]. That is why ctDNA can be considered “real-time” snapshot to reflect the overall evolution of lesions [12]. This enables real-time and long-term monitoring of the treatment effect, allowing feasible treatment adjustment and better prognosis. Moreover, ctDNA facilitates dynamic monitoring of the clonal evolution and helps to identify the emergence of resistant subclones [12]. Up to now, the European Medicines Agency [27] and the FDA [28] have approved epidermal growth factor receptor (EGFR) mutation testing using ctDNA for therapy guidance among non-small cell lung cancer (NSCLC) patients. Moreover, some newly approved immunotherapies are known to produce different tumor response patterns from other systemic treatments. Using the regular practice of monitoring therapeutic efficacy might not be appropriate anymore. For patients treated with immune checkpoint inhibitors for NSCLC, ctDNA was shown to be an early marker of therapeutic efficacy and could better predict survival outcomes [29].

### Residual disease and risk of relapse

Even when treatment is successful, relapse is still a significant threat to many cancer patients, and it is hard to detect the residual disease in time using imaging or tissue biopsy. Very few effective and reliable markers are available currently. Recent studies demonstrated that ctDNA assays were able to detect residual disease several weeks earlier than radiologic imaging [30], and ctDNA-positive patients were at higher risk of relapse and exhibited worse outcome (like shorter overall survival and disease-free survival time) compared to the ctDNA-negative group [31]. In addition, it was shown that phylogenetic ctDNA profiling could be used to track the subclonal nature of lung cancer relapse and metastasis [15]. With the collected profiles, cancer patients could be stratified into different adjuvant therapies to prevent overtreatment [12].

## Sequencing techniques

The concentration of ctDNA in plasma has been shown to correlate with tumor size [32] and stage [33]. Patients having stage I disease with various cancer types had fewer than 10 copies per 5 ml of tumor mutations in plasma. In contrast, the copy number increased 10 to 100 times among late-stage patients [34]. Thus, ctDNA assays used for early cancer diagnosis should be highly sensitive. However, highly sensitive assays are always expensive, making large-scale practical applications unrealistic. For late-stage cancer tumor typing, the sensitivity can be moderate because the concentration of ctDNA is much larger. At the same time, the cost of the ctDNA assay is acceptable, and there are several commercial platforms available (Table 1). There is always a tradeoff between sensitivity and cost. Various methods have been proposed to reduce cost, background noise, and errors induced in the amplification step. Those methods can be categorized in different ways. Based on the techniques, there are PCR-based sequencing and NGS-based sequencing. Based on the assay panel size, there are single-locus/multiplexed assays, targeted sequencing, and genome-wide sequencing. PCR-based sequencing can be used for single-locus/multiplexed assays and targeted panel, while NGS-based sequencing can be applied to any panel size.

**Table 1:** List of liquid biopsy companies

Company	Product/study	Usage/aim	Reference
Grail	The SUMMIT Study	Evaluating a blood test designed to detect multiple types of cancer, including lung cancer	[35]
Guardant 360	Lunar-2	Early cancer detection among higher-risk asymptomatic individuals	[36]
Freenome	Screening test	Using AI-based algorithm for early detection of colorectal cancer and precancerous lesions known as advanced adenomas	[37]
Biocept	Target Selector™ ctDNA EGFR Kit	EGFR mutation detection	[38]
Inivata	InVisionSeq™ and InVisionFirst™- Lung	Around 40 biomarkers (including mutations, CNV, SNV, fusions, indels) panel for advanced cancers molecular profiling, monitoring, and diagnosis	[39]
Cynvenio	The LiquidBiopsy® Platform	NGS-based techniques to detect mutations in as few as 1 target cell per mL/blood	[40]
CellMaxLife	FirstSightCRC™	Using circulating tumor cells for colorectal cancer, adenomas, and colorectal cancer screening and profiling	[41]
Exosomedx	Exosome-based biomarker tests*	Diagnosing non-small cell lung cancer and prostate cancer	[42]
Biodesix	GeneStrat® test	Providing blood-based mutation results of EGFR, ALK, ROS1, RET, BRAF, and KRAS for cancer diagnosis	[43]
Personal Genome Diagnostics	PlasmaSELECT™ -R 64	Cancer diagnosis using NGS with 64 Genes panel	[44]

*Exosomes are extracellular vesicles (EVs) produced by cells. They contain RNA, proteins, lipids, and metabolites that are reflective of their origins [45]

### PCR-based methods

PCR-based methods are most widely used and can achieve extremely high sensitivity. PCR-based methods can be divided into three major categories: real-time quantitative PCR (qPCR), digital PCR (dPCR), and the mass-spectrometry-based method. qPCR is commonly used since it is fast and relatively inexpensive [46]. However, it can only detect mutant allele fraction (MAF) that is greater than 10% [47]. Several variations have been developed to improve the sensitivity of qPCR. For example, co-amplification at lower denaturation temperature (COLD-PCR) can preferentially amplify mutant sequences by controlling the denaturation temperature. It was proved to be a robust method to detect MAF of approximately 0.1% [48, 49].

dPCR has a similar principle as qPCR except it partitions the sample into thousands of parallel PCR reactions to reduce background noise. Thus, it can detect MAF that is less than 0.1% [50]. The sensitivity can be further enhanced by using multiplexed patient-specific panels [51] or molecular barcoding [52] to reduce background sequencing error rates. Among variants of dPCR, BEAMing (on the basis of four of its principal components: beads, emulsion, amplification, and magnetics) is considered to be the most sensitive approach with the detection rate of 0.02% [53]. Nevertheless, the protocol is complicated, and it is relatively expensive for routine clinical usage. It uses primer-bound beads to combine DNA template and distribute the mix in oil detergent to create many aqueous compartments that contain no more than one template or bead. Then, the whole system undergoes conventional PCR. Since each template is distributed into a separated reaction space, the amplification of template is more specific and fewer errors are induced. In the end, fluorescent hybridization and flow cytometry are applied to distinguish and count different templates.

Apart from qPCR and dPCR, the mass-spectrometry-based method is an adaptation of the conventional PCR method with a unique advantage in multiplex detection. For example, UltraSEEK can detect mutant sequence mixtures with MAF as low as 0.1%. It first applies multiplex PCR to amplify all mixtures at the same time. Then, mutations are captured with the labeled chain terminators for sing-base extension and identified using matrix-assisted laser desorption/ionization time-of-flight mass spectrometry [54].

### NGS-based methods

Although PCR-based methods are sensitive and inexpensive, they can only screen for known variants, and the input and speed are limited. NGS has high throughput and can screen unknown variants. Currently, NGS is able to detect MAF *<* 1% [55]. Furthermore, many methods like unique molecular identifiers [29] or unique barcodes [16] can help to increase the sensitivity and reduce the false negatives. These methods are able to detect 59% of stage I or II lung cancer patients with MAF around 0.1% [16] and have good agreement between ctDNA response and radiographic response [29]. NGS can be applied to the targeted panel for specific and highly sensitive detection of targeted ctDNA mutations. Many methods are applying NGS to target panel, namely Tagged-Amplicon deep sequencing (TAm-seq), Safe-Sequencing System (Safe-SeqS), CAncer Personalized Profiling by deep sequencing (CAPP-Seq), and Ion Torrent.

For TAm-seq, researchers first design special primers to amplify regions of interest. In order to control sampling errors and allelic loss, the primers are first used to bind to the template during a preamplification step to amplify the original signal. Next, the templates undergo individual amplification for purification. Benefiting from this two-step amplification design, TAm-seq may able to identify mutations ~ 2% MAF with sensitivity over 97% [56]. The enhanced version of TAm-Seq, named eTAm-Seq™ can detect MAF as low as 0.25% with a sensitivity of 94%. In addition, it has been revised to identify single-nucleotide variants (SNVs), short insertions/deletions (indels), and copy number variants (CNVs) [57].

For Safe-SeqS, the key idea is adding a unique identifier (UID) to each template. After amplification, if a mutation does not appear in most of the same UID-connected sequences, it is likely to be induced by other errors. In this way, Safe-SeqS reduces the sequencing errors by at least 70-fold [58] and has sensitivity as high as ~ 98% for detecting tumor mutations [59].

CAPP-Seq is a combination of the library preparation method and a specialized bioinformatics workflow. The library generates many hybrid affinity captures of recurrently mutated genomic regions from the population of interest to create a “selector.” The “selector” is applied on tumor DNA to identify individual-specific mutations as prior knowledge. Then, it is applied ctDNA for quantification [52, 60]. CAPP-Seq can detect MAF ~ 0.02% with a sensitivity of nearly 100% among stage II-IV NSCLC patients [61].

Ion Torrent is an NGS platform developed by Thermo Fisher Scientific. It allows CNVs, single-nucleotide polymorphisms (SNPs), indels, and fusion detection with as little as 1 ng DNA input [62]. One study applied this platform covering 2800 COSMIC (the Catalogue Of Somatic Mutations In Cancer) mutations from 50 cancer genes to successfully identify 71% of metastatic breast cancer patients [63]. Another study covered more than 6800 COSMIC mutations of 46 genes. About 97% of mutations identified in metastasis biopsies were detected in matched ctDNA in the study [64]. However, researchers who compared dPCR with Ion Torrent concluded that dPCR was more sensitive and can detect smaller MAF for some targeted panels [65].

Although targeted panels may be preferred for their high sensitivity and low cost, they can only detect point mutations and indels. One unique advantage of NGS is that it can be applied to the untargeted panel to find genome-wide DNA variation. Whole-genome-sequencing (WGS) is usually used to get the whole genomic profile of tumor DNA including point mutations, indels, rearrangements, and CNVs [46]. Although WGS provides us with abundant information, it is expensive and less sensitive. Whole-exome sequencing (WES) is a popular alternative of WGS. It is less expensive by only sequencing the exons. Nevertheless, both WGS and WES require high input sample volume, hindering their application in screening and early diagnosis when the concentration of ctDNA is considerably low. Many genome-wide sequencing methods have been proposed for different variation types like PARE (personalized analysis of rearranged ends) for the detection of rearrangement, digital Karyotyping for DNA content quantification, and FAST-SeqS (Fast Aneuploidy Screening Test-Sequencing System) for the detection of CNVs.

PARE first uses next-generation mate-paired sequence analysis to identify individualized rearrangements from tumor tissue. Then, it applies PCR for quantitatively monitoring the detected rearrangements. It is highly sensitive for detecting ctDNA lower than 0.001% in patient plasma samples [66]. Some studies suggested that ctDNA at levels *>* 0.75% could be detected in cancer patients with sensitivity over 90% and specificity over 99%. Even a single copy of rearrangement from ctDNA can be detected without false positives [67].

Digital karyotyping is a quantitative approach to detecting genome-wide abnormalities at high resolution, including unknown chromosomal changes, altered regions, and DNA sequences [68]. It uses two enzymes to cut the DNA into short fragments around 10 kb and ligates each fragment with a tag. The tags help to align the DNA fragments back to the genome and detect abnormalities in DNA sequence through their density. Orthodenticle homologue 2 (OTX2) amplification was identified in medulloblastomas using digital karyotyping. The overexpression of OTX2 was later confirmed to be causal for certain medulloblastomas type [69].

FAST-SeqS (Fast Aneuploidy Screening Test-Sequencing System) can discriminate as low as 4% of trisomy 21 DNA from euploid samples. The key is simplifying the library preparation steps by only using one designed single primer pair to amplify the repeat regions of interest, so that the cost can be controlled while increases the throughput [70]. There is an updated version of FAST-SeqS called modified FAST-SeqS (mFAST-SeqS). Unlike methods like PARE for quantification of target mutations predetermined by sequencing tumor tissue, mFAST-SeqS is an untargeted method to monitor residual disease or treatment response. Compared with the targeted approaches that can detect MAF as low as 0.01% to 0.5%, untargeted approaches can only detect MAF *>* 10%. Nevertheless, untargeted approaches require no prior knowledge and can develop genome-wide copy number pattern or assess mutation spectra [71, 72].

### Methylation sequencing

Cancer screening not only requires knowing whether the person has cancer or not, but also needs to find the cancerous site for follow-up diagnosis and treatment. Somatic mutation alone may not provide adequate information about the tumor site. Epigenetic information like methylation [73] or protein biomarkers combined with ctDNA [74] has been proved to help determine the tumor origin at an early stage. It is especially useful when the primary site of cancer is unknown. Researchers found that the tumor- and-tissue-specific pattern from methylome data can help with disease classification [75, 76]. It has been shown that methylation profiles of hepatocellular carcinoma tumor DNA and matched plasma ctDNA were highly correlated [75] and could be used to differentiate breast, colon, liver, and lung cancer in diagnosis and prognosis [77].

Methylation sequencing techniques usually have a preprocessing step before sequencing. In addition to DNA conversion, the intention of the preprocessing step is enriching and selecting sequencing targets to reduce the cost. For example, some protocols use immunoprecipitation against 5-methylcytosine to allow much lower levels of input DNA while maintaining high sensitivity [73, 78]. In some other cases, methylation-sensitive restriction enzymes are used to analyze DNA methylation changes [79, 80].

Similar to DNA variants detection, the limited concentration of methylation variants poses great challenges for the balance between coverage, cost, and sensitivity while controlling the technical errors introduced during sequencing. Various methods have been proposed to address the trade-off. For example, the locus-specific techniques like methylation-specific PCR [81] and MethyLight [82] can achieve high sensitivity. However, they can only provide semi-quantitative information for a particular pattern of DNA methylation. PCR-based target selection can achieve high accuracy with a low level of input [81, 83]. However, it cannot be easily applied to the whole-genome level. On the other hand, bisulfite sequencing facilitated by NGS [61, 84, 85] can achieve genome-wide coverage. Adoptions of bisulfite sequencing like Padlock probes can enrich arbitrary target set [86], and DREAMing can detect ultra-rare heterogeneously methylated epialleles variants [87].

## Challenges

### Biological challenges

ctDNA is highly fragmented, ranging from 100 to 10,000 bp. It is challenging to isolate ctDNA from the blood for quantitation since the small fragments are easy to lose or degrade [88]. Although the concentration of ctDNA will increase with the stage and tumor size, the total percentage of ctDNA in the blood is extremely low, putting many requirements on the sample processing procedure. Also, it has been shown that both concentration and stability of ctDNA could be influenced by the form, release, degradation, and clearance of cfDNA [89]. Up to now, very few studies have discussed the clearance rate and biological mechanism of ctDNA. Another significant obstacle at present is the lack of biological knowledge and experimental evidence to support the quantitative relationship between ctDNA and early cancer development. The pathological evidence is hard to find. Since by the time of using the ctDNA assay for cancer screening or early detection, no knowledge of tissue samples or symptoms of cancer is available. Much remains for us to understand the fundamental biology of ctDNA before we can further push forward the clinical applications of liquid biopsy.

### Panel design

It is challenging to find the optimal panel of biomarkers (in most cases, this refers to genetic mutations) according to different objectives, which may demand different tests and impose different requirements [22]. For example, screening requires high sensitivity and high coverage, while monitoring will focus more on the specificity of given mutations. Traditionally, the candidate gene mutation panel is decided on limited biological or clinical knowledge. Nowadays, bioinformatics and biostatistics tools are broadly used to guide the panel design. Information from databases like COSMIC [90] or The Cancer Genome Atlas (TCGA) [91] can be integrated to find differential expression genes or cancer-related mutants among cancer patients and healthy controls. Nevertheless, published studies often applied different methods to select the mutation panels, and there are no systematic criteria on how to choose the optimal combination.

Recently, some researches combined ctDNA mutations with other biomarkers like protein or methylation to improve the overall sensitivity. It was shown that the combination of ctDNA and protein biomarkers could dramatically increase the sensitivity [17]. However, it could be difficult to find the optimal combination of other biomarkers that can maximize the overall detection performance. Biostatistical approaches allow us to effectively identify the relationship between biomarkers like the correlation pattern to guide the panel selection. For example, a study has shown that using *KRAS* mutations with four protein biomarkers can increase the sensitivity from 30 to 64% and *TP53* provided little improvement to the panel since it was highly correlated with *KRAS* [74].

### Sample processing

In a recent review [92], it was proposed that the pre-analytical sample processing including collection, handling, transport, processing, and storage of a specimen is crucial to the final result of ctDNA assay since they would increase degradation of cell-free DNA or increase contamination. The recovery of smaller DNA fragments is particularly important in ctDNA analyses. Many approaches have been explored to improve sample processing quality. For example, plasma has been proven to be the superior source of ctDNA [93]. Standard lavender top tubes with anticoagulant EDTA are most suitable for sample collection [94, 95]. To conclude, a standard operating procedure for ctDNA pre-analytical sample processing is essential to allow more robust and comparable results. However, many published studies were retrospective studies and used archived serum or plasma with distinct pre-analytical procedures [96–98]. Little is known so far on how those variables would influence the accuracy of the test.

### Data analysis

ctDNA sequencing, especially using NGS, will produce large amounts of data. In addition, in the context of disease monitoring, repeated measurements of clinical variables and outcomes and sequencing data will be collected. The large data size and complex date challenge to statistical analysis. First, researchers need to decide on the lower limits before conducting tests. However, the optimal lower limits of detection may vary depending on the intended use of the ctDNA assay, and there are no standard criteria for choosing the lower limits [99]. Some articles found that ctDNA was highly concordant with tumor DNA while others did not [100–102]. It was suggested that the discordant results might depend on the genetic tests applied [100] apart from variation in biosource.

Another statistical challenge is building the classification model. Since the sample size is usually small compared to the number of biomarkers, selecting a subset of most important biomarkers helps to avoid overfitting. Different methods have been used for biomarker selection and model training in published studies [17, 75]. However, some of the procedures were not appropriate. For example, one of the most commonly seen mistakes is using all data for model training and testing, which might induce bias and appear to have high accuracy. Although many model selection methods are available, without appropriate training, testing, model comparison, and diagnosis procedure, the results could be biased and invalid.

The third problem is how to integrate data from different resources. This is especially challenging for cancer screening where we can collect longitudinal data regarding ctDNA sequencing, other biomarkers like protein and methylation, demographic data, medical record, living habits, and so on. Combining available information can help distinguish different populations and improve diagnostic accuracy. A model like CancerSEEK uses both mutation data and protein data to achieve high classification accuracy [17]. One drawback of CancerSEEK is that it transforms all ctDNA mutation data into a single omega score and puts it into the model with other protein biomarker data instead of directly using all the information contained in ctDNA mutations. There are few methods available to build such a model that can integrate different data types, track the change over time with suitably selected predictors, and maximize the usage of all available information.

### Clinical applications

Two paradigms are proposed for demonstrating clinical validity and utility using ctDNA [92]. First, prospective clinical trials can be used to test ctDNA as an independent test. Alternatively, the information provided by ctDNA and tissue samples can be assessed to compare their similarity. Both paradigms face many challenges especially in the context of disease screening and early diagnosis. For the first one, the validation of the assay quantitation of tumor burden is technically challenging due to the sample processing issues discussed above. In addition, absolute quantitation is hard to obtain. Most of the methods only obtain relative measures, and few studies conducted cross-platform comparisons. Even when the accurate measure can be obtained, clinical validation requires large-scale prospective trials including both healthy people and cancer patients for treatment guidance and outcome evaluation.

For the second paradigm, the concordance between tumor tissue and ctDNA are not consistent across different studies. A significant number of studies showed that the correlation between plasma mutation status and response rates to therapy was almost the same as that of tumor tissue [103–108]. Nevertheless, other studies showed covariates such as disease stage, tumor type, and tumor heterogeneity and whether the variant was clonal or subclonal could influence the concordance between tissue and plasma mutation status [21, 92, 109, 110]. These observations suggest that although it is necessary to develop the concordance between tissue and ctDNA, directly relating ctDNA mutation profiles to clinical measurements of cancer may be another strategy. Last but not least, there is the concern of false positives and overdiagnosis brought by cancer screening. Some patients will not become symptomatic, or their tumors can be benign even they are tested positive. Whether the benefits overwhelm the additional cost and the medical pressure brought by the practice of liquid biopsy remains to be carefully examined [111].

Up to now, there are many liquid biopsy-based assays designed for disease detection, diagnosis, profiling, and treatment selection. Some of them have already been used commercially on cancer patients (Table 1). However, most studies about liquid biopsy were observational, and some of them lacked healthy controls. To date, no studies have shown any improvement in patients outcomes or medical cost using liquid biopsy compared with standard-of-care monitoring methods [92]. Moreover, few studies have evaluated the treatment outcome only based on ctDNA assay-guided targeted therapy. Few of previous studies were intended for cancer screening and early diagnosis. Nevertheless, many large-scale prospective studies are undergoing to demonstrate the clinical validity and utility of ctDNA assays rigorously. For example, powered by Illumina, a company named GRAIL planned to start the SUMMIT study enrolling approximately 50,000 participants without cancer from a high-risk population. They aimed to develop an affordable blood test to detect multiple types of cancer at the same time (Table 1).

## Conclusion

Up to now, ctDNA has shown many promising results for cancer classification, monitoring, prognosis, and treatment selection. However, using ctDNA for cancer screening and early detection remained to be solved. The biggest challenge is the low concentration of ctDNA in the blood. Although some NGS-based protocols improve the sensitivity of ctDNA assays in many different ways, the trade-off between sensitivity and cost is still the greatest concern in practice. In the future, other sources of information apart from ctDNA should be combined to increase sensitivity and specificity. Moreover, applying ctDNA sequencing to cancer screening provides us with a good opportunity to collect longitudinal data to create a better disease classification model. As the price for sequencing continues to decrease, using liquid biopsy for cancer prevention and treatment hold promise in the future.

## Data Availability

Not applicable

## Abbreviations

CA: Carbohydrate antigen
CAPP-Seq: CAncer Personalized Profiling by deep sequencing
cfDNA: Cell-free DNA
CNV: Copy number variant
COLD-PCR: CO-amplification at Lower Denaturation temperature
COSMIC: The Catalogue Of Somatic Mutations In Cancer
CRC: Colorectal cancer
ctDNA: Circulating tumor DNA
FAST-SeqS: Fast Aneuploidy Screening Test-Sequencing System
FDA: The US Food and Drug Administration
indel: Insertion or deletion
MAF: Mutant allele fraction
mFAST-SeqS: Modified FAST-SeqS
NGS: Next-generation sequencing
NSCLC: Non-small cell lung cancer
OTX2: Orthodenticle homologue 2
PARE: Personalized analysis of rearranged ends
PCR dPCR: Digital PCR
PCR TAm-seq: Tagged-Amplicon deep sequencing
qPCR: Real-time quantitative
Safe-SeqS: Safe-Sequencing System
SNP: Single-nucleotide polymorphisms
SNV: Single-nucleotide variant
TCGA: The Cancer Genome Atlas
UID: Unique identifier
WES: Whole-exome-sequencing
WGS: Whole-genome-sequencing
